# The evolution of healthcare through the eye: From ancient superstition to the ophthalmoscope

**DOI:** 10.3389/fmed.2026.1775739

**Published:** 2026-03-03

**Authors:** Alexander Pinhas

**Affiliations:** Department of Ophthalmology, Icahn School of Medicine at Mount Sinai, New York, NY, United States

**Keywords:** healthcare through the eye, history of medicine, history of ophthalmology, ocular biomarkers, ophthalmoscopy, retinal biomarkers, systemic disease detection, preventive medicine

## Abstract

The eye has emerged as a vital window into systemic health and a powerful tool for proactive, preventive healthcare. This study explored how ideas from antiquity to the 19^th^ century gradually led to the emergence of ophthalmoscopy and the contemporary model of healthcare through the eye. Key developments across the Eurasian continent were examined through the lenses of cultural anthropology, historical medicine and ophthalmic science. Texts were selected to illustrate major conceptual transitions concerning disease causation and the perceived relationship between the eyes and internal organs. As early as ancient Sumeria in the 3rd millenium BCE, people interpreted changes in the external appearance of eyes as signs of demonic possession of internal organs, viewing the eyes as conduits for entering and exiting spirits, and employing talismans to counter supernatural harm caused by the evil eye. Between 500 BCE – 200 CE, the Greek *Hippocratic Corpus*, the Chinese *Huang Di Nei Jing*, and the Indian Ayurvedic writings gradually replaced demonic and godly explanations with rational yet inaccurate models of disease involving flows of humors, jing-qi, and doshas, respectively. These rational yet inaccurate models retained certain motifs from the prior irrational models, including extramission theories of vision, and ideas of external eye signs revealing disturbances within the body. These frameworks were able to advance empirical thinking despite their inaccuracies, allowing later evidence-based breakthroughs including Ibn al-Haytham’s proofs of intromission, Galvani’s discovery of electrical conduction in nerves, and Harvey’s demonstration of a closed circulatory system. The invention of the ophthalmoscope by Helmholtz in 1851 marked a decisive turning point, enabling clinicians for the first time to directly visualize the retina and optic nerve in living patients. With ophthalmoscopy, the eye was transformed into a true diagnostic window for systemic disease, firmly establishing the eye’s modern role in systemic health assessment. These findings suggest that the modern diagnostic use of the eye for internal health reflects a deep historical trajectory from superstition to rationalization to scientific validation. Understanding this evolution provides context for current interest in ocular biomarkers and systemic disease detection through imaging, helping to inform culturally sensitive approaches to population health.

## 1 Introduction

Healthcare through the eye refers to the long-standing idea that the eye is connected to internal health and well-being. This concept is extraordinarily ancient, appearing in some of the earliest written records across civilizations. Today, we recognize this concept as being scientifically valid and backed by evidence-based medicine, and we understand many of the physiological mechanisms behind it. However, for most of human history, the association between the eye and internal health appears to have been intuitive, with interpretations of this relationship being shaped by various irrational and rational misconceptions.

As an eye clinician engaged in retinal imaging and the evaluation of systemic disease through ocular findings, I have repeatedly encountered situations in which retinal or optic nerve changes prompted alterations in the systemic management of neurological, cardiovascular, or metabolic disease. In discussing these findings with patients, I have noticed that the concept of the eye as a reflection of internal health resonates with patients from diverse ethnic groups and geographic backgrounds, often aligning with pre-existing cultural beliefs. I have found that this cultural familiarity helps facilitate patient understanding and acceptance of ocular screening for systemic disease.

These observations indicated to me that the development of healthcare through the eye as an applied medical practice reflects not only scientific and technological advancement, but also the influence of culturally-mediated perceptions of health and disease. The present work emerged from an effort to situate modern ocular diagnostics within their deeper historical and conceptual lineage within the field of medicine. By examining how early cultures understood the relationship between the eye and systemic disease; and, by tracing how these ideas evolved through the lenses of cultural anthropology, historical medicine, and ophthalmic science, we may gain valuable insight into how the eye became globally regarded as a diagnostic window into the body. Such analysis may also support more historically- and culturally-informed implementation of healthcare through the eye as a population-wide approach to preventive medicine.

This review follows key milestones from antiquity to the invention of the ophthalmoscope in 1851, highlighting pivotal transitions from superstition to observation and early scientific reasoning, laying the groundwork for the eye’s modern role in systemic health.

## 2 Methods of literature search

A narrative literature review was conducted to identify historical and scientific sources relevant to the evolving understanding of the eye as a diagnostic window to systemic disease. Because this review is focused on highlighting pivotal moments rather than providing a comprehensive history, sources were selected based on their ability to illustrate key inflection points in thought, practice, or technology. Source selection was guided by three primary criteria: (1) relevance to conceptual models linking ocular findings with internal health, (2) historical influence on medical or ophthalmic thought, and (3) availability of authoritative primary texts or peer-reviewed secondary analyses. Searches were performed in MEDLINE/PubMed, EMBASE, and Google Scholar, covering the time period from antiquity through the end of the 19^th^ century. Search words used included combinations of:

“eye” AND “symbolism” OR “superstition
“eye” OR “retina” AND “internal medicine” OR “systemic disease
“eye” OR “retina” AND “systemic biomarkers” OR “oculomics
“ophthalmoscope” OR “ophthalmoscopy” AND “history” OR “Helmholtz
“retinal imaging” AND “noninvasive diagnosis
“history of medicine” AND “eye” OR “vision
“history of medicine” AND “circulatory system” OR “cardiology
“history of medicine” AND “nervous system” OR “neurology

Publication years covered in the search included Jan 2000–December 2025. Reference lists of key articles and historical reviews were examined manually to identify additional relevant sources not indexed in the primary databases. Peer-reviewed journal articles and scholarly books were included if they contributed to understanding pivotal transitions in how the eye has been used to interpret systemic health across history. Sources were included if they described cultural, medical, technological, or conceptual turning points that redefined the role of ocular examination in systemic disease detection. Mostly English-language articles and English-language abstracts were included. If relevant foreign-language articles were encountered, key sections were interpreted using language support tools.

Exclusion criteria included non-peer-reviewed sources and editorial opinions; articles not directly relevant to pivotal transitions in understanding the eye’s systemic role; foreign-language sources without accessible abstracts; publications focused solely on the eyes or on technical aspects of examining the eyes without systemic relevance; and, redundant reviews or secondary sources without original insights.

In addition to peer-reviewed journal databases, historical texts were identified and accessed through specialized digital archives, including the Wellcome Collection, Internet Archive, and Google Books, which host digitized 19th-century ophthalmic literature. Publication years covered in this search included 1851–1900 (the half century following the invention of the ophthalmoscope). These sources were selected for their primary relevance to key historical developments in retinal diagnostics and the invention of the ophthalmoscope. Many of these sources were originally published in German or other non-English languages. Where available, English translations or summaries were consulted; otherwise, key sections were interpreted using published English abstracts, modern translations, or language support tools. These texts were included due to their foundational historical significance, despite not being indexed in standard biomedical databases such as MEDLINE or EMBASE.

## 3 Narrative review

### 3.1 Superstitious believes in early antiquity

#### 3.1.1 Spirits entering the body through the eyes cause disease

For thousands of years, cultures across the ancient world, including Mesopotamia, Egypt, Greece, India, and China explained disease through the actions of spirits, demons, and angered gods (1). The eye, understood as more than just an organ of sight, was widely regarded as a doorway through which spirits could enter and exit the body (2). Upon death, the proper closure of eyes was ensured, at times with metallic eyepatches, to prevent ghosts of dead people from re-entering and re-animating their deceased bodies (3). In the living, disease-causing spirits, once inside the body, were believed to afflict specific organs and functions (4). The eyes were thought to retain connections to afflicted organs, and changes in the external appearance of the eyes were interpreted as outward signs of internal organ possession (5).

Within this construct, one of the earliest recorded ocular biomarkers of internal disease was recognized by the ancient Sumerians as the yellowing of the eyes, thought to be secondary to demonic possession of the soul, which resided in the liver. As early as the third millennium BCE, Cuneiform tablets representing diagnostic medical texts from the ancient Sumerian and Akkadian civilizations described the clinical signs of ahhazu, or jaundice (5, 6). These tablets read “If his face is yellow and the inner part of his eyes is yellow, and the base of the tongue is dark, ahhazu;” and, “If ahhazu rises to a person’s eyes so that his eyes are covered with a network of yellow threads, his insides are puffed up and return bread and beer to his mouth, if that person lingers, he will die.” The demon that was possessing, or seizing, the soul residing in the liver, and thus causing ahhazu, was aptly named Ahhazu (4). Perhaps the saying “the eyes are the window to the soul” has its roots in the yellow appearance of eyes in people with liver disease, whose souls were thought to be possessed by demons.

Other examples from Sumerian records of internal spiritual possessions manifesting in ocular signs included (5):

“If he was injured on his head and, as a consequence, his eyes are heavily clouded, hand of Ningirsu.”

“If his right armpit hurts him and his eyes are clouded, hand of Lamashtu.”

“If his epigastrium is hot and the blood vessels of his temples, his hands and his feet continually feel like they are pulsating and his eyes feel heavy, affliction by a ghost.”

“If a burning pain is firmly established in his abdomen on the left side and his eyes are full of yellow threads, hand of Ishtar; he will die.”

“If his insides are continually bloated and his eyes feel heavy, hand of Kubu; he will get well.”

“If in the middle watch, he keeps crying out: ‘My insides, my insides,’ his eyes are confused and his eyesight is difficult and his left foot continually afflicts him, anger of a rabisu continually afflicts him; he will speedily die.”

“If his limbs are supple, his epigastrium has a needling pain, blood incessantly flows from his nose, his arms are continually weak, depression continually falls upon him and his eyes are suffused with blood, hand of Marduk; he is in danger of dying.”

**Figure 1:**
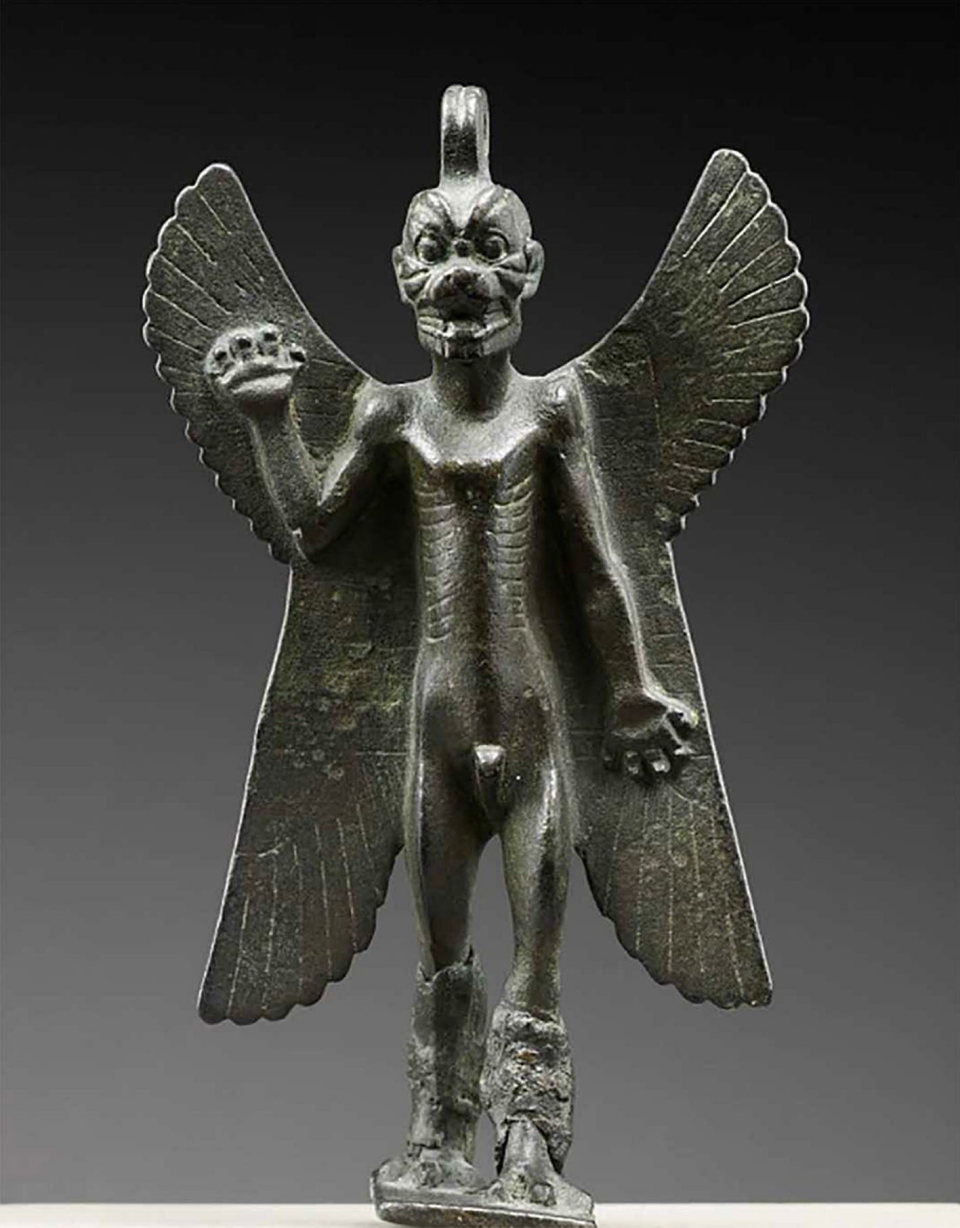
An idol of the demon named Pazuzu was used as an apotropaic device and during exorcisms in Mesopotamia as early as the 1st millenium BCE. Pazuzu’s gaze was a powerful weapon that could be leveled against that of other demons, especially Lamashtu. Borrowed with permission from Konstantopoulos (8).

Treatments of disease were comprised of divinations and exorcisms using various herbs and animal parts including animal internal organs, which were designed for ridding the patient’s internal organs of demonic possession, and not necessarily for their pharmacological benefits (7). Treatments often focused on the patient looking at a consecrated object, in order for the object to lure the bad spirit back out of the body through the patient’s eyes and to capture it, or in order for the object to defeat the bad spirit with its own gaze, ridding the patient of the bad spirit (Figure 1) (8).

#### 3.1.2 Envious eyes emit a malevolent gaze causing curse and disease

The “evil eye” is among the oldest and most widespread superstitions in human history, with roots extending across the ancient Middle East, India, the Mediterranean, Africa and Latin America. The belief centers on the idea that a malevolent gaze itself, originating from the soul (liver or heart) and exiting through the eyes, especially one driven by envy, can bring about harm, illness, misfortune, or even death. The belief dates to as early as the third millennium BCE in Mesopotamia, referenced by Sumerian and Akkadian clay tablets and protective amulets (9).

**Figure 2:**
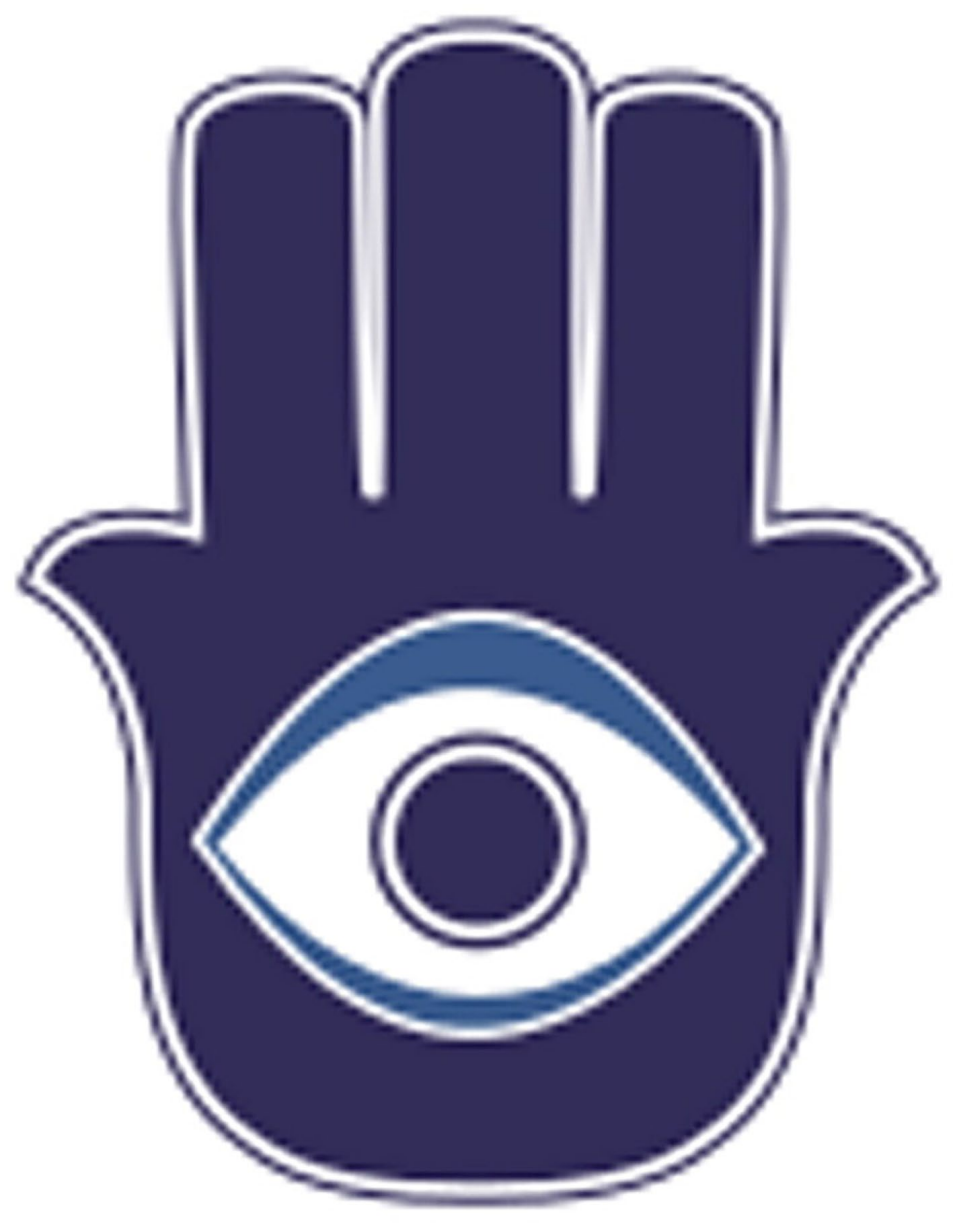
This Hamsa hand contains an eye-shaped object to ward off the evil eye. Borrowed from Kopel (11) (open access).

To guard against the perceived danger of human or non-human curse-emitting eyes, early civilizations fashioned eye-shaped objects and idols with over-sized eyes as protective talismans for both people and property (2). This practice marked one of the earliest symbolic links between the eye and internal well-being. Since eyes were viewed as conduits through which the spiritual forces moved in and out of the body, eye-shaped talismans were believed to intercept the harmful essence emitted by a hostile gaze. By drawing the malevolent force into the object itself or repelling it in some way away from its intended target, the talisman served as an apotropaic spiritual shield against the destructive power of the evil eye (10). Another theory held that eye-shaped talismans were believed to be evil eyes themselves, emitting a more powerful harmful essence that overpowered the harmful essence emitted by the hostile gaze (2). Remarkably, the superstitious practice of the evil eye endures to this day in many cultures, where eye-shaped amulets and Hamsa hands continue to be worn or displayed to protect people and property (Figure 2) (11).

### 3.2 Reorientation toward rational thought in early antiquity and classical antiquity

**Figure 3:**
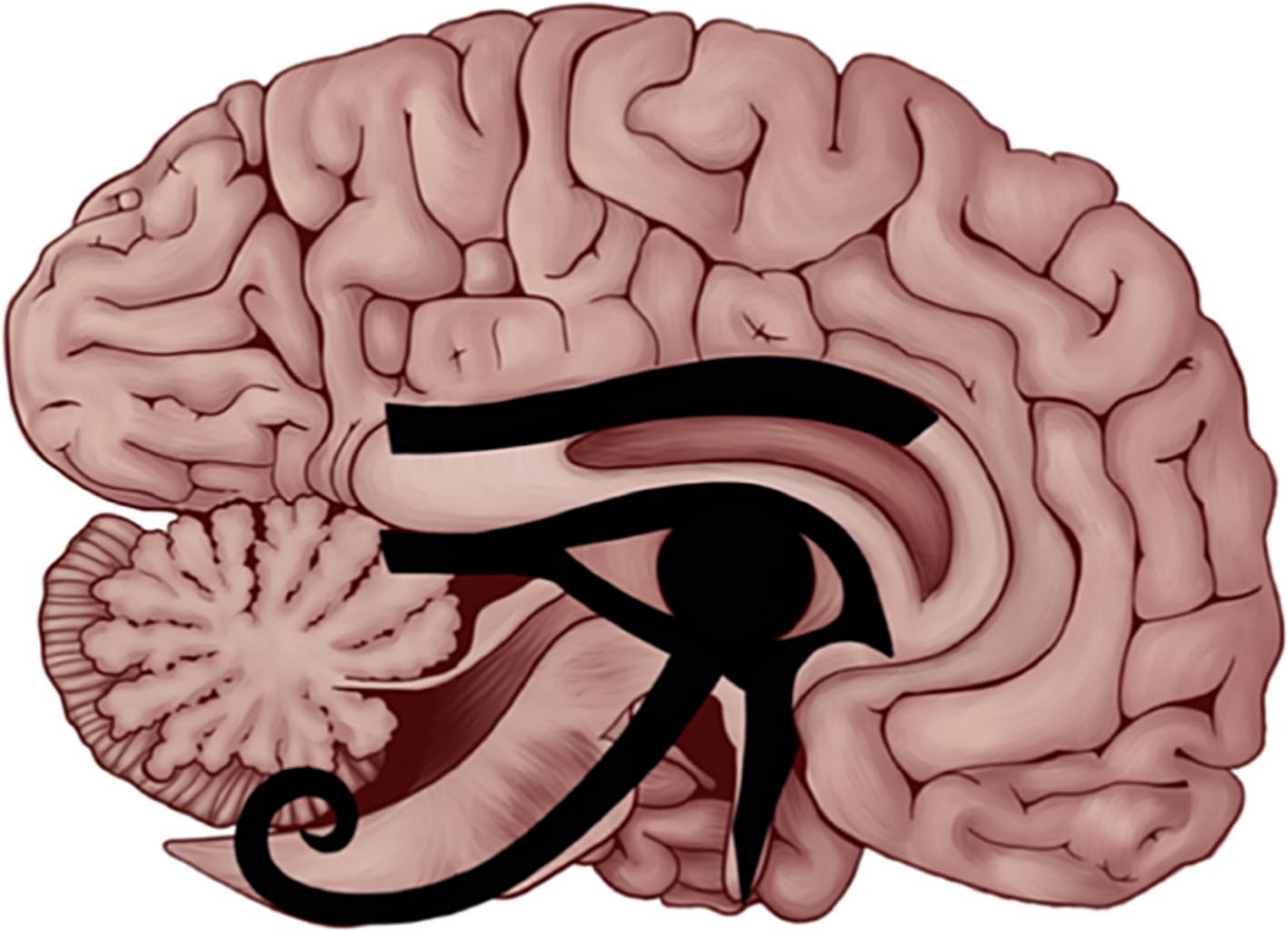
The eye of Horus fitted onto the mid-sagittal section of the human brain. Different parts of the eye of Horus fit the anatomical structures that carry special brain function including smell, vision, wisdom, hearing, taste, and touch. Borrowed from ReFaey et al. (12) (open access).

In ancient Egypt, as early as 2,700 BCE, the Eye of Horus, beyond its widely recognized role as a protective and healing symbol, may have held deeper anatomical significance. The image may have been intentionally designed to resemble the human brain and its sensory pathways, particularly those associated with smell, vision, wisdom, hearing, taste and touch. When overlaid on a mid-sagittal section of the brain, various elements of the Eye of Horus appear to correspond to structures such as the corpus callosum, thalamus, hypothalamus, and optic chiasm. This alignment has led scholars to propose that the Eye of Horus functioned not only as a mythological or superstitious emblem, but also as an early symbol for a more rational connection between the eye and brain (Figure 3) (12).

The notion that people can emit harmful influence through their eyes likely contributed to the extramission theory of vision and other extramission-like theories, which held that vision was an outward phenomenon involving flow from the eyes that somehow interacted with external objects allowing the observer to see (10, 13). Evidence suggests that some form of extramission theory existed across several major ancient civilizations by the 5th century BCE, including Greece, India and China (14–16). Extramission and extramission-like theories created a conceptual pathway linking the appearance of the eyes to internal organ function, since flows believed to originate within internal organs and flowing upward and outward through the eyes would result in changes to the appearance of the eyes.

**Figure 4:**
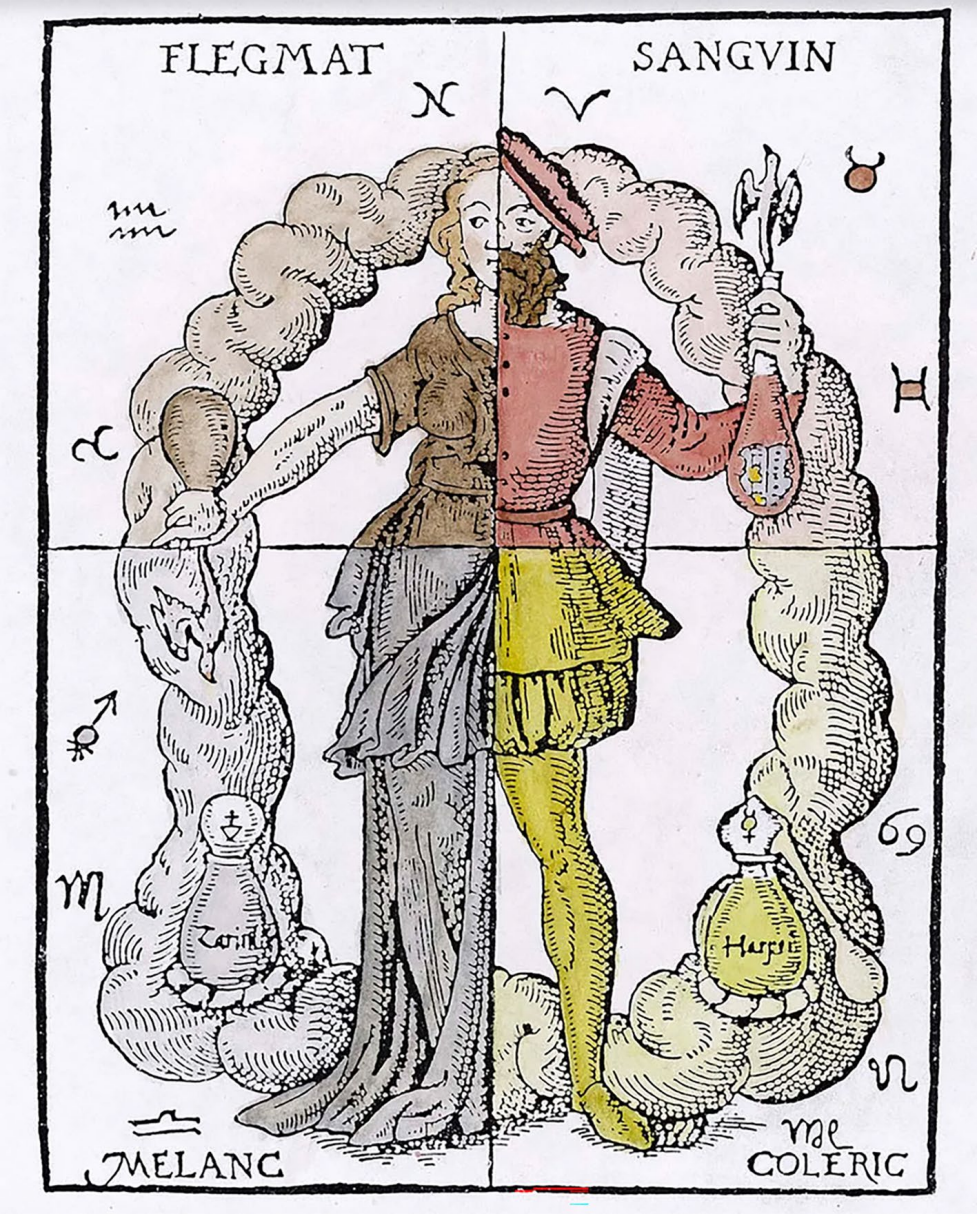
The four humors, according to Hippocrates. Borrowed from Thurneisser zum Thurn (18) (public domain).

In the Western world, Hippocrates is credited as the first person to write that diseases were caused naturally, and not because of superstitious entities or godly punishments. Attempting to rationally explain the pathogenesis of disease, the *Hippocratic Corpus*, a collection of Greek medical writings compiled between the 5th and 3rd centuries BCE, was based on Hippocrates’ theory that the body contained four humors, namely blood, phlegm, black bile and yellow bile (Figure 4). These four humors flowed through and nourished the body and its internal organs, and disease resulted from alterations in their equilibrium, and other factors such as environment, diet and patient habits. During diseased states, imbalances in the humors reached the surface of the body and manifested as color changes of the skin and the sclera of the eyes. In the case of jaundice and scleral icterus, excess yellow bile affected liver function and produced the yellow discoloration of the skin and eyes. Inflammation or redness in the eyes was interpreted as an excess of blood humor, prompting bloodletting as a treatment (17, 18). Thus, while the rational yet inaccurate construct of the *Hippocratic Corpus* replaced flows of demonic spirits with the flows of humors, it maintained the eye’s central role as an indicator of internal disease.

**Figure 5:**
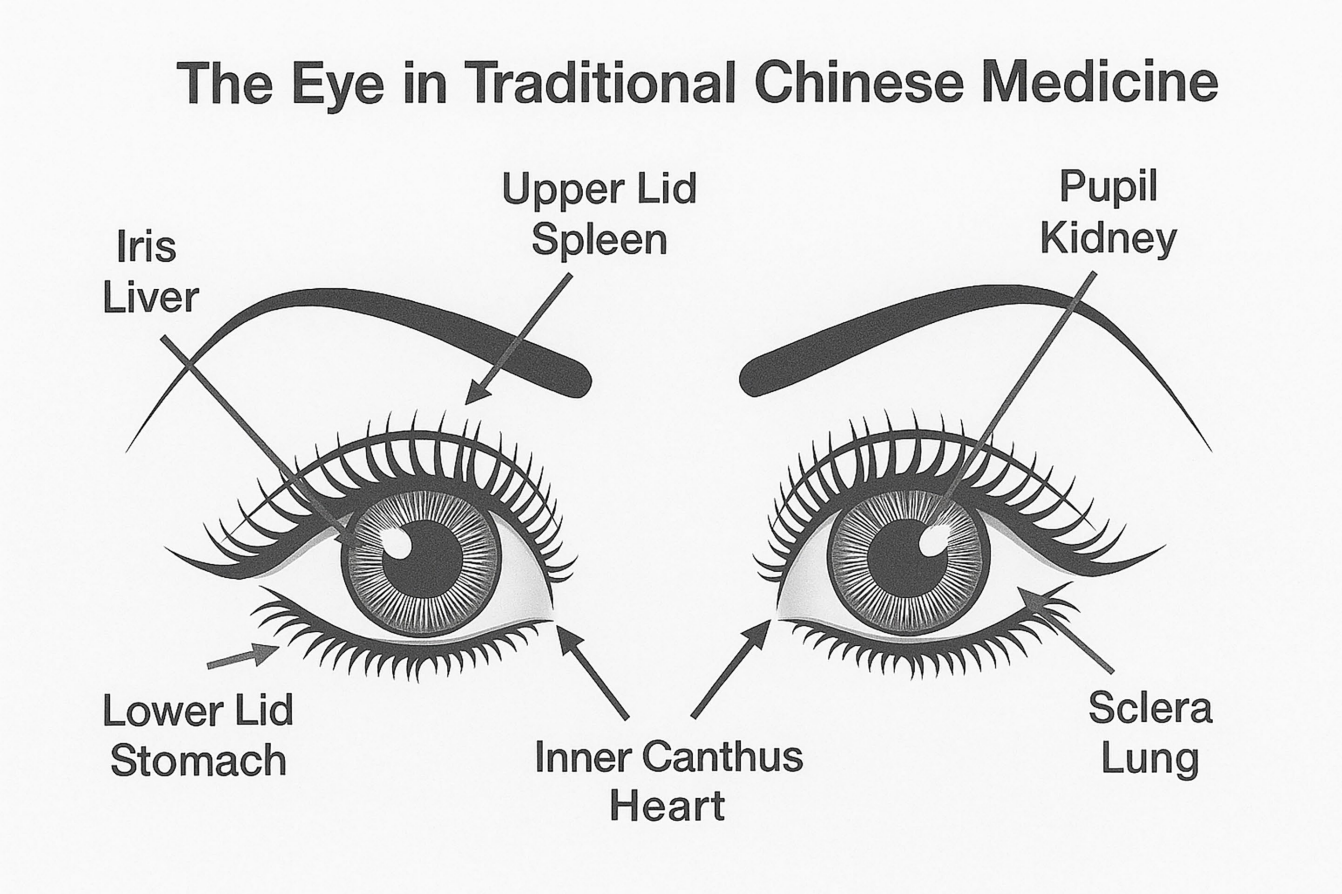
Eye diagnostics in traditional Chinese medicine. The *jing-qi* flowed from a given internal organ to corresponding parts of the eyes.

In China, the *Huang Di Nei Jing* (the *Yellow Emperor’s Inner Canon*), compiled during the Warring States period (475–221 BCE), represented the foundation for traditional Chinese medicine and strove to be a rational work. Unlike earlier Chinese works, and similar to the *Hippocratic Corpus*, the *Huang Di Nei Jing* took pride in explicitly rejecting old shamanistic beliefs, the influence of spirits and the use of magic (19). The *Huang Di Nei Jing* posited that the jing (essential life force or essence) and qi (vital energy) of the internal organs flowed upwards and out through the eyes, enabling the eyes to see. It followed that through this flow of jing-qi, lesions of the internal organs revealed themselves in the eyes, namely in the eyes’ expression, color, shape and movement. The eye was thus leveraged as an adjunct method to judge the condition of internal organs. Each internal organ corresponded to a specific part of the eye, namely the liver to the iris, the kidneys to the pupil, the lungs to the sclera, the heart to the inner and outer canthi, and the stomach to the eyelids (Figure 5) (20).

In India, around the same general period as the *Hippocratic Corpus* and *Huang Di Nei Jing,* Ayurvedic medicine developed a naturalistic framework centered on the three doshas (humors), which gradually supplanted earlier Vedic explanations grounded in divine and demonic causation (21). By the 2nd century CE, classical compendia such as the Charaka Samhita and Sushruta Samhita identified the small intestine as a principal seat for pitta dosha (bile), the colon for vata dosha (wind), and the chest for kapha dosha (phlegm), and taught that pitta dosha was primarily responsible for visual perception. Disturbances in these doshas were understood to produce systemic disease, which often manifested in characteristic changes in ocular appearance and function (22). Thus, classical Ayurveda too tended away from superstitions, while preserving the eye as a crucial site for recognizing presence of internal organ disease.

**Figure 6:**
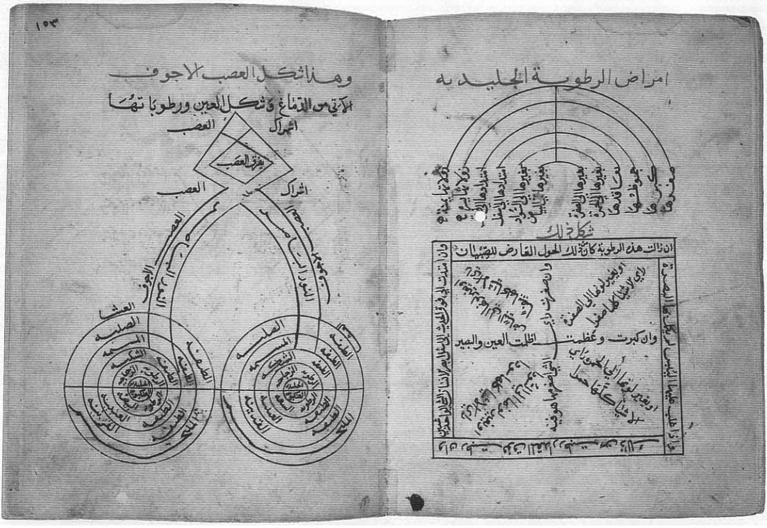
A diagram of the visual system from *De morbis oculorum*, Galen’s book on diseases that occur in the eye. The original is lost, and this remaining Arabic copy by Abd al-Rahman ibn Ibrahim ibn Salim ibn Ammar al-Maqdisi al-Ansari al-mutatabbib [the physician] dates to 1,160 AD. Borrowed with permission from Savage-Smith (23).

Galen, a Greek physician in the Roman Empire and student of the *Hippocratic Corpus*, proposed that other eye signs can be used as part of the physical exam to detect internal disease, including how the eyes felt on palpation (hot, cold, hard), their size, the nature of their movements and their function. His studies on eye anatomy from the second century CE distinguished the cornea, sclera, lens, retina, choroid, extraocular muscles, and the connection between the optic nerves and the brain (23). Galen’s physiological mechanism for extramission proposed that psychic pneuma, a specific life force under the influence of the soul and produced in the brain, reached the eyes through small hallow channels in the optic nerves, made its way to the lens through hallow retinal blood vessels, was emitted from the lens and then recaptured by the lens as it bounced back from objects, giving the eye its ability to see. He believed that the lens, and not the retina, was the most important part of the eye responsible for visual sensation (24). Galen further postulated that the blending of returning psychic pneuma within the optic chiasm gave rise to binocular vision (Figure 6) (23). Serving as an early rational but inaccurate model for the connection between visual acuity and the brain, decreased vision was interpreted as a weakened production of psychic pneuma secondary to a poorly functioning brain.

### 3.3 Toward the real in the middle ages and modern period

#### 3.3.1 Intromission and scientific revolutions

The definitive scientific rejection of extramission came from Ibn al-Haytham, whose groundbreaking treatise, Kitāb al-Manāẓir (Book of Optics), completed by 1021 CE during the Islamic Golden Age, presented a rigorous mathematical and experimental case for intromission. Ibn al-Haytham was the first scholar to conclusively demonstrate that light did not emanate from the eye, as ancient theories claimed, but instead originated from external sources, reflected off objects, and entered the eye. He moreover identified the retina, and not the lens, as the principal organ of visual reception, proposing that visual impressions were conveyed via the optic nerves to the brain, where visual perception occurred. He also theorized that the brain, and not the optic chiasm itself, integrated the input from both eyes, resulting in binocular vision (25).

**Figure 7:**
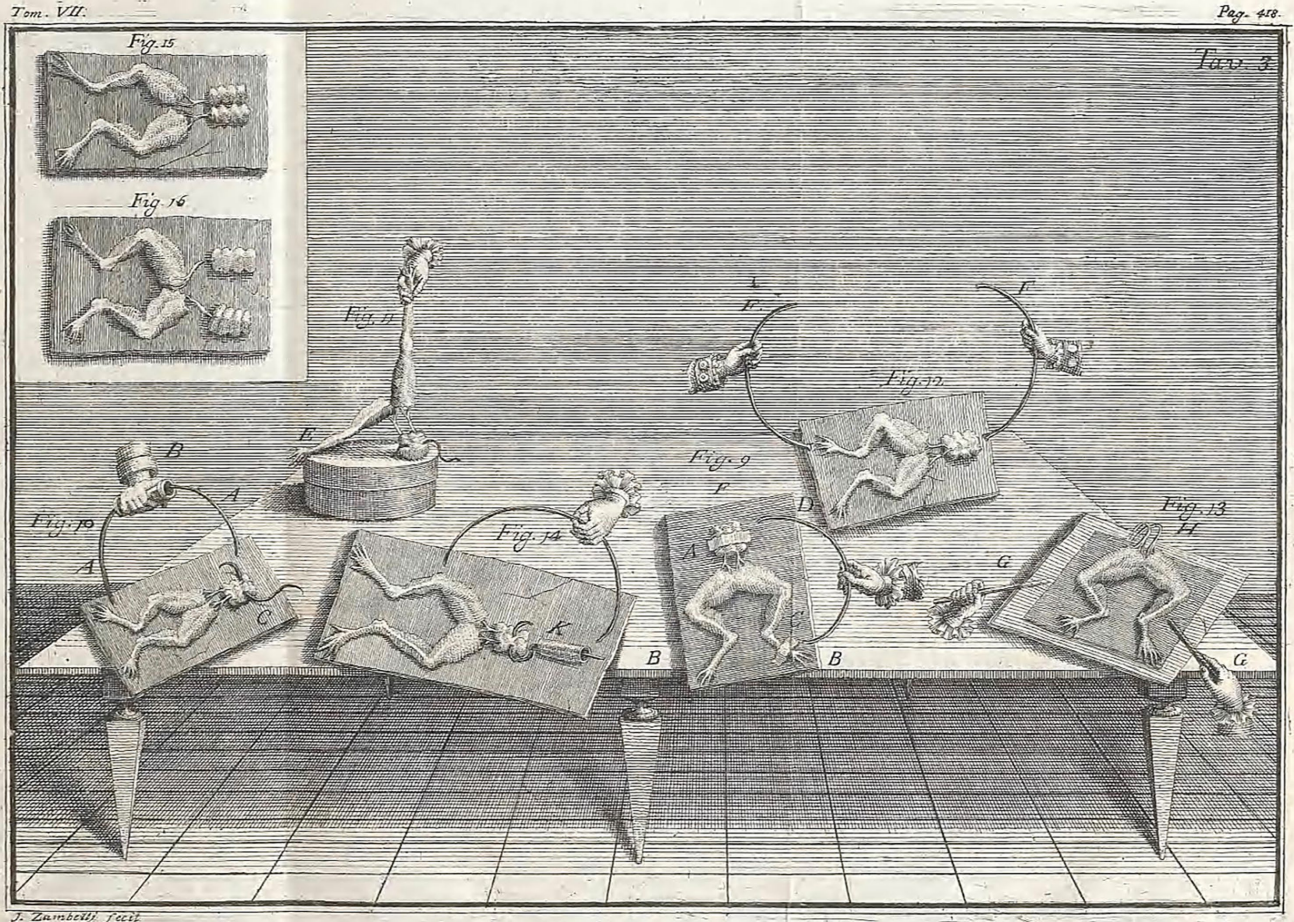
An illustration of Galvani’s frog experiments, borrowed from Galvani (26) (public domain). Galvani used external electrodes to stimulate muscle contractions in deceased frog limbs.

It wasn’t until Luigi Aloisio Galvani’s nerve electricity proposal in 1791 that Galen’s theories of nerve pneuma were abandoned. Galvani proposed that the cortical material of the brain generated an electrical fluid, and not psychic pneuma, and that it was this electrical fluid that flowed through hallow nerves (Figure 7) [(26), p. 66]. These were the beginning notions of an electrical nervous system, and it wasn’t until Edgar D Adrian’s experiments in the 20th century that it was realized that the conduction of electricity through nerves resulted from the transfer of ions across nerve fiber cell membranes (27).

**Figure 8:**
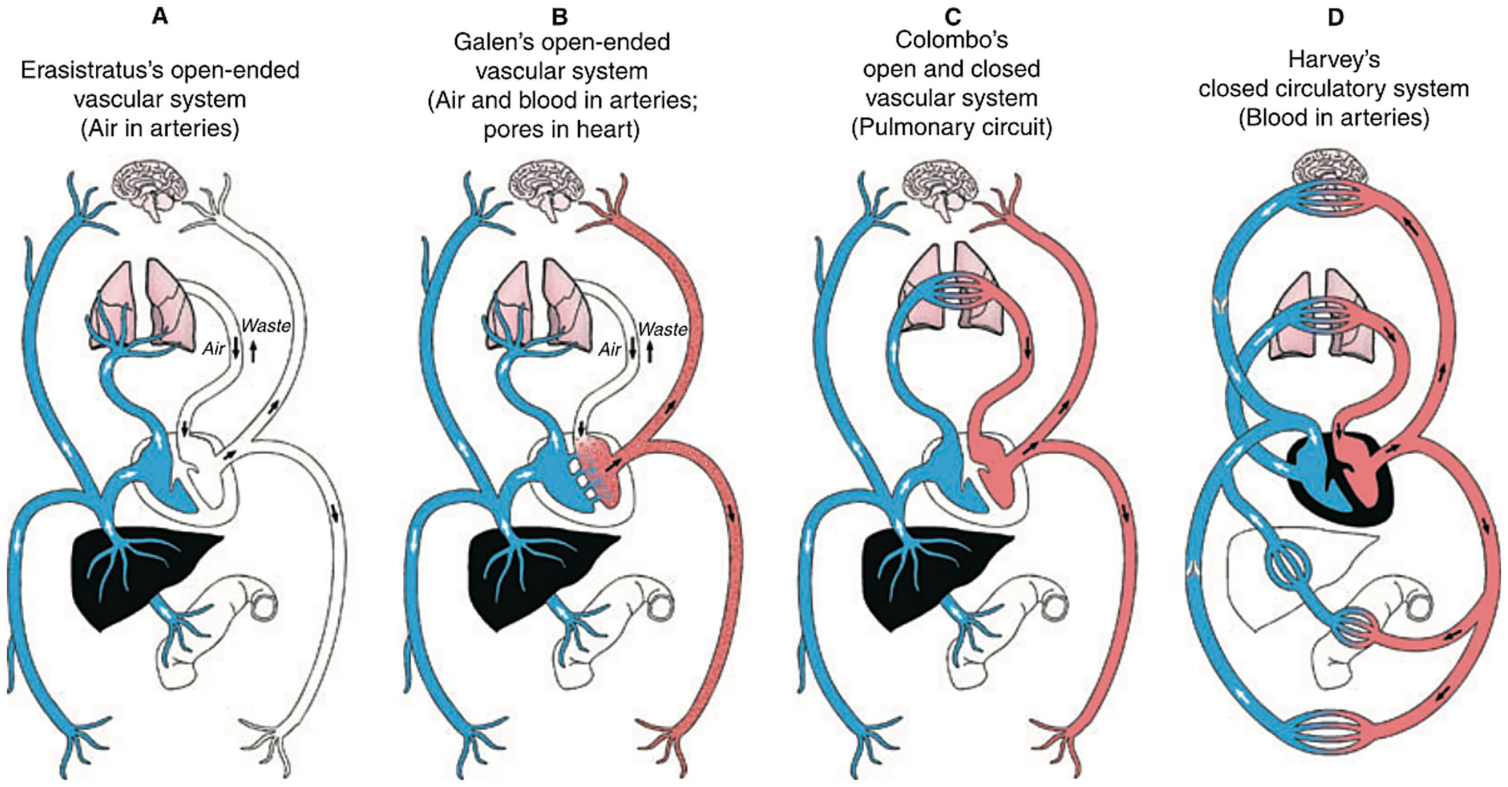
Diagrams comparing Galen’s **(B)** and Harvery’s **(D)** blood flow models, among others. Borrowed with permission from Aird (28).

And it wasn’t until William Harvey’s blood circulation proposal in 1628 that Galen’s theories of blood and air flow were likewise abandoned. Galen had proposed that the cardiovascular system was comprised of two distinct networks of arteries and veins. Blood produced in the liver reached the right heart via veins, and continued from the right heart via veins to nourish the body, never returning to the liver or heart. Air inhaled by the lungs reached the left heart via pulmonary veins, blood from the right heart also entered the left heart via small pores in the interventricular septum and mixed with air, and this mixture of blood and air continued from the left heart via arteries to nourish the rest of the body, likewise never returning to the heart or lungs. These open-ended systems allowed blood and air to simply dissipate at the ends of veins and arteries into the peripheral tissues. Harvey challenged these views with experiments and deductive reasoning, and argued that arteries and veins are connected in the lungs and peripheral tissues, and that blood circulates (Figure 8) (28).

#### 3.3.2 Iridology: a modern revival of an ancient idea

**Figure 9:**
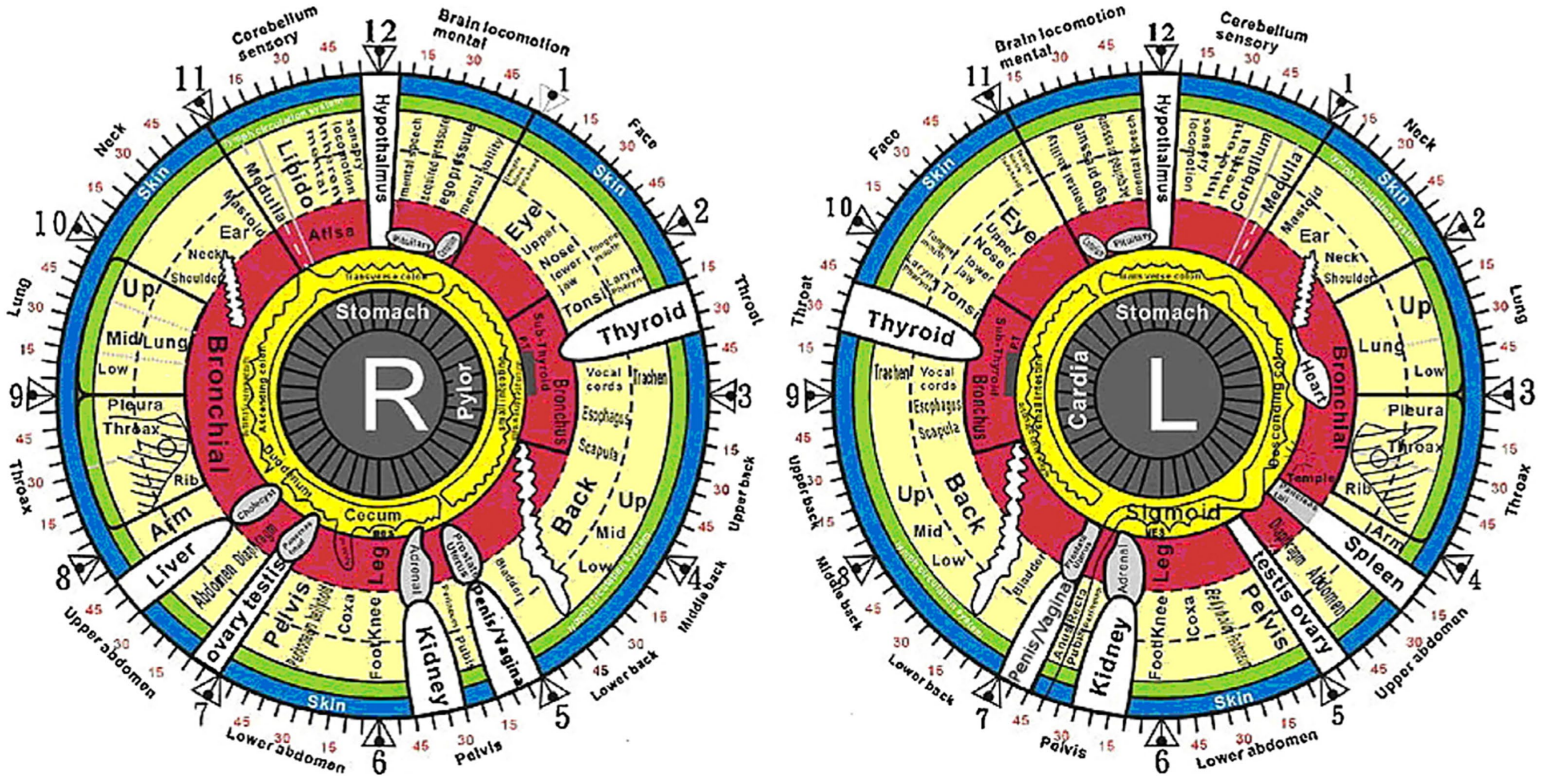
The iridology chart for both the right and left irises. Borrowed with permission from Hussein et al. (29).

Later physicians sought to expand on the connection between external eye features and internal health. The field of iridology, focusing on the correlation between iris appearance and systemic disease, represented a modern revival of the pre-modern concept that internal organ essence radiates to the eyes. Iridology was introduced by German physician Philip Meyen von Coburg in 1665 and formulated by Hungarian physician Ignaz von Peczely in 1861. Peczely proposed that specific zones of the iris corresponded to different organs and systems in the body, and that localized changes in the iris, such as iris pattern or color, indicated the presence of corresponding internal organ dysfunction (Figure 9) (29). Although iridology represents one of the earliest attempts in modern society to link ocular features to systemic health, the majority of it currently lacks scientific validity (30).

#### 3.3.3 Ophthalmoscopy: the breakthrough that enabled true healthcare through the eye

*“In the whole history of medicine there is no more beautiful episode than the invention of the ophthalmoscope, and physiology has few greater triumphs… by its means we are able to look upon the only nerve in the whole body which can ever lie open to our inspection under physiological conditions…”*

-Ophthalmologist Edward Greely Loring of New York in the opening paragraph of his Textbook of Ophthalmology in 1892, two years before the death of Hermann von Helmholtz (27, 31).

**Figure 10:**
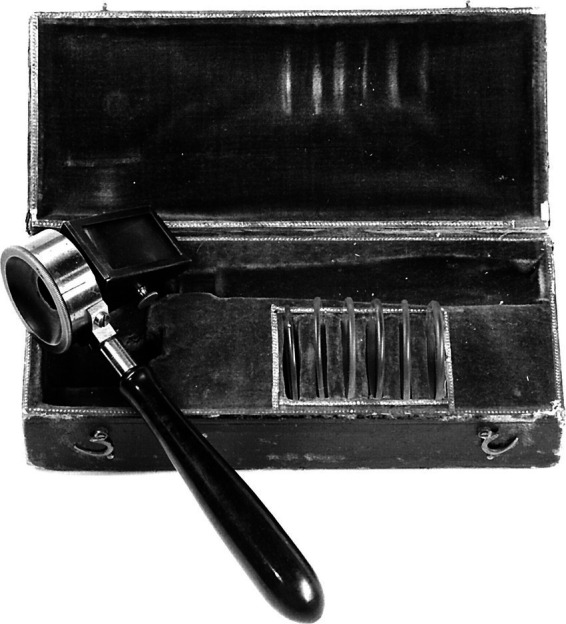
An early model of the Helmholtz ophthalmoscope, 1851. Borrowed with permission from Keeler (34).

Hermann von Helmholtz’s invention of the direct ophthalmoscope in 1851 marked a pivotal breakthrough in healthcare through the eye, enabling, for the first time, direct visualization of the posterior pole in the living patient, a region previously inaccessible to the naked eye yet highly relevant to systemic health (32). Before the invention of the ophthalmoscope, the retina remained largely inaccessible to clinicians. Even post-mortem histological study offered little insight, as the retinal layers degenerated rapidly after death and early 19th-century techniques were unable to preserve or visualize them reliably (33). The invention of the ophthalmoscope allowed scientists and clinicians to observe the optic nerve and retina as extensions of the brain and the retinal vasculature as a reflection of the cardiovascular system in the living patient (32). The innovation combined three essential components: a source of illumination (initially a flickering candle), a reflective surface to direct light into the eye, and an optical mechanism to correct the out-of-focus light reflected from the posterior pole as it exited the eye (Figure 10) (34).

Thus, the bridge between rational misconception to reality was finally crossed. After the invention of the ophthalmoscope, physicians and scientists across Europe rapidly adopted its use and began making contributions to the understanding of optic nerve and retinal diseases and their connection to the central nervous system and the cardiovascular system (32). Von Graefe’s pioneering studies using the ophthalmoscope between the 1850s and 1860s provided insights into ocular manifestations of systemic disease, including optic neuritis in multiple sclerosis, optic disc swelling in increased intracranial pressure, central retinal artery occlusion caused by emboli from endocarditis, and choroidal changes associated with tuberculosis (31).

**Figure 11:**
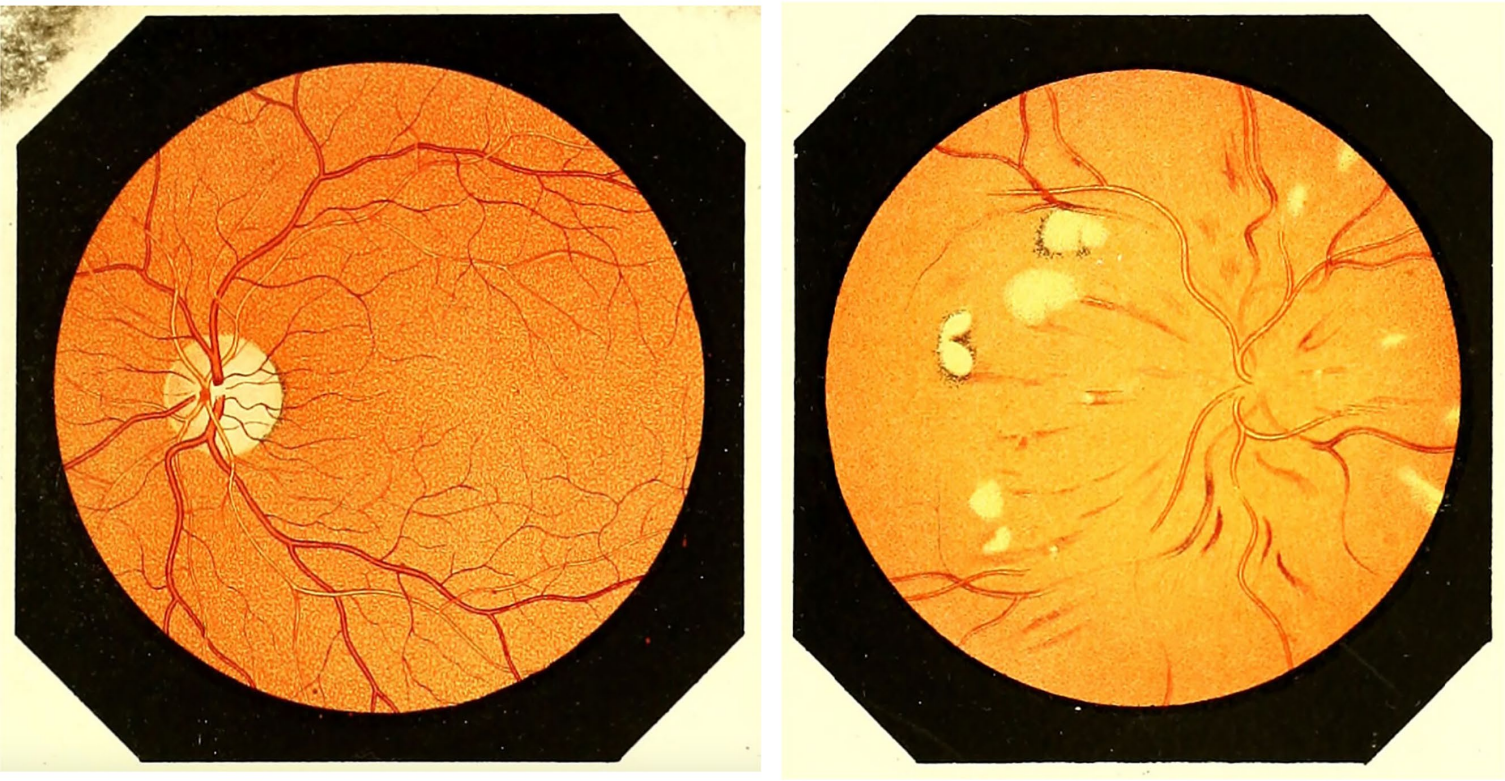
From Jaeger’s book Ophthalmoscopic Hand-Atlas, left drawing shows a healthy retina and right drawing shows retinal changes in the setting of diabetes mellitus. Borrowed from Jaeger (38) (public domain). Figures 23 and 64 in the book.

**Figure 12:**
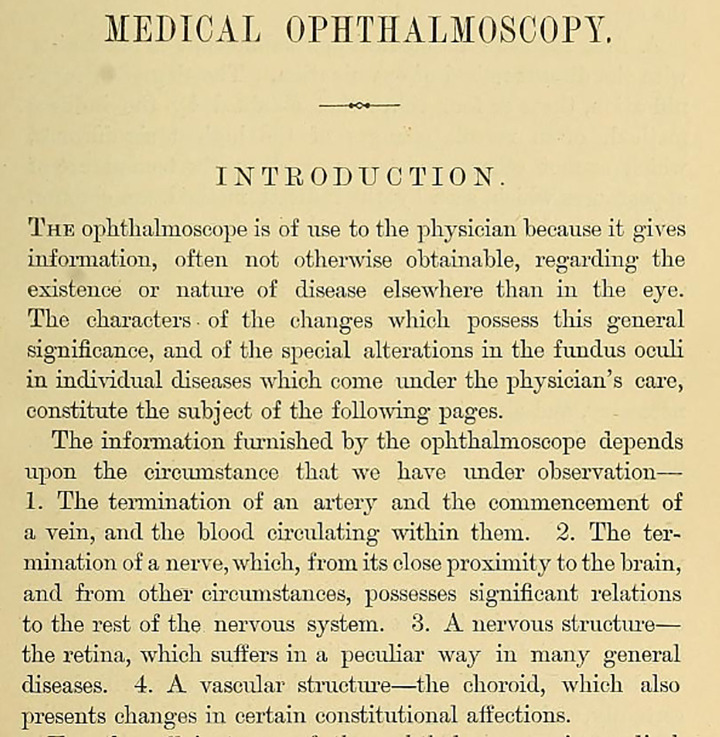
The introductory paragraphs of Gowers’ Medical Ophthalmoscopy: A Manual and Atlas written in 1879. Borrowed from Gowers (37) (public domain).

**Figure 13:**
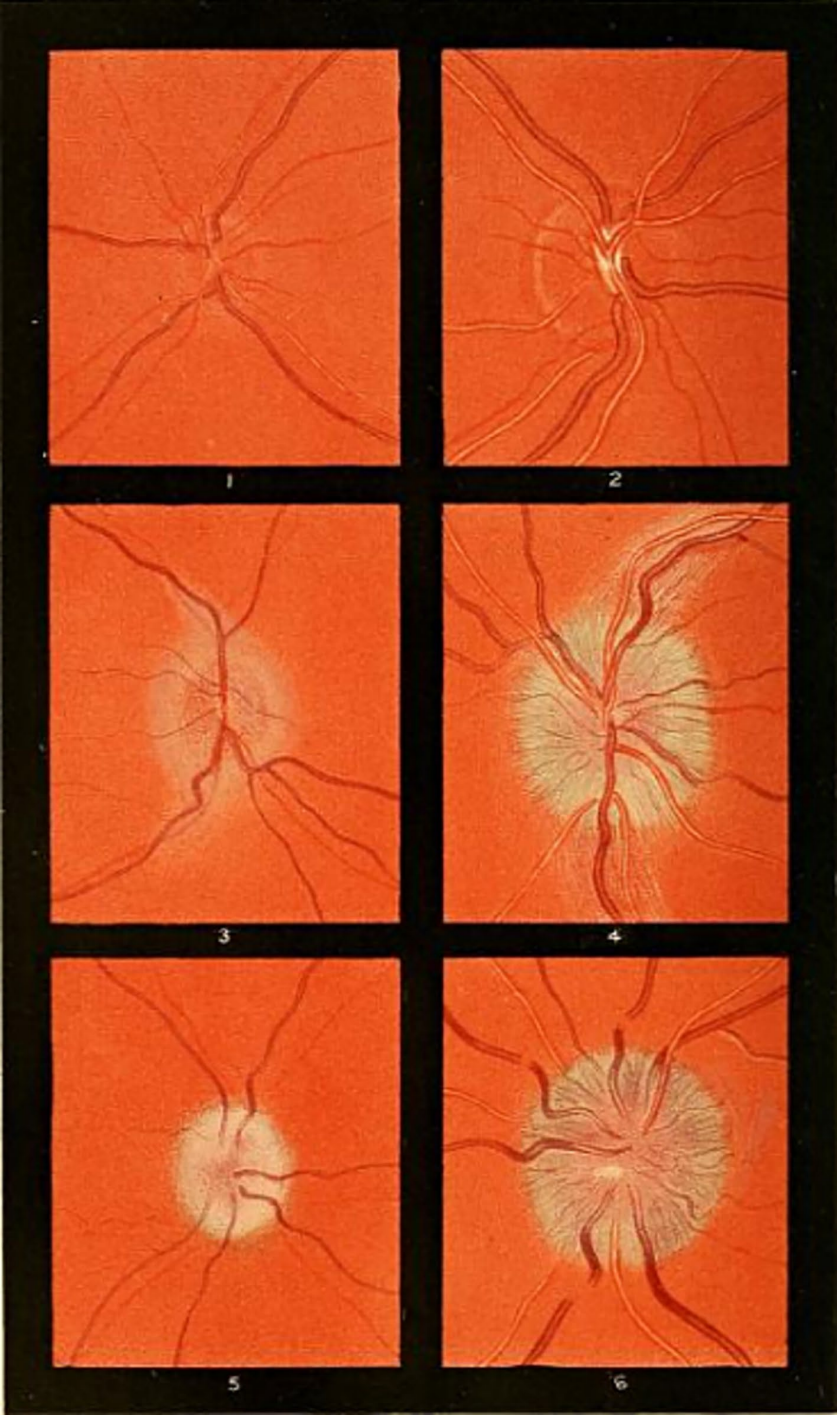
Examples of optic nerve head drawings from Gowers’ book. (1, 2) Some mild edema of the right optic nerve head in a patient who suffered large right cerebral stroke, with resulting left hemiplegia. (3, 4) Large edema of the left optic nerve head in a patient with neurosyphilis. (5, 6) Large edema of the right optic nerve head in a patient with an intracranial tumor. Borrowed from Gowers (37) (public domain).

Other contributors included Liebreich who in 1859 linked retinal changes to chronic kidney disease (35), and Becker who in 1869 highlighted retinal hemorrhages and other retinal findings in patients with leukemia (36). Jaeger in 1869, Leber in 1877 and Gowers in 1879 wrote comprehensive books detailing observable retinal and optic nerve changes in various systemic conditions including diabetes mellitus, hypertension, neurological disorders, hematological disorders, and infectious diseases (Figures 11–13) (37–39).

In 1898, Marcus Gunn further elaborated on retinal vascular changes in patients with hypertension and kidney disease, writing that the retinal arteries “have an exceptionally bright reflex; the central light-streak is very distinct and sharp, while the whole surface of the vessel is of a somewhat lighter color than usual,” and posited that “…such visible primary changes in retinal arteries are very commonly, if not constantly, a part of a similar, more general, and therefore more important change in small arteries elsewhere.” He also remarked that when such a diseased retinal artery “passes over a retinal vein, the circulation of the latter is much impeded” (40). Marcus Gunn realized that one of the important manifestations of systemic hypertension was changes to the retinal vasculature, laying the groundwork for studying the most common chronic medical condition through the eye.

## 4 Discussion

As early as the 3rd millennium BCE, many ancient cultures viewed the eye as a portal through which spirits could enter or exit the body, and changes in the external appearance of eyes were read as signs of disease-causing demons seizing internal organs. Beliefs surrounding the evil eye, along with the widespread use of protective talismans against it, further underscored the perceived centrality of the eye to internal well-being.

By the 5th century BCE, however, major medical traditions had begun to abandon supernatural explanations of illness. Both the *Hippocratic Corpus* in the West and the *Huang Di Nei Jing* in the East rejected demonic possession and divine punishment as causes of disease, adopting instead rational though still inaccurate explanatory models. Yet, these systems retained certain motifs, including extramission theories of vision, and the idea that the outward appearance of the eyes could reveal disturbances within the body.

The rejection of superstition and embrace of rational yet incorrect models helped establish empirical observation and scientific reasoning as the legitimate path forward in medical explanation, allowing later evidence-based breakthroughs including Ibn al-Haytham’s proofs of intromission, Galvani’s discovery of electrical conduction in nerves, and Harvey’s demonstration of a closed circulatory system. With the invention of the ophthalmoscope in 1851, the journey of healthcare through the eye transitioned from ancient superstitions and rational misconceptions toward an era of empirical science. Early ophthalmoscopy and retinal illustrations definitively established as true the ancient intuition that the eye is not merely an organ of sight, but a uniquely accessible window into systemic health.

A deeper understanding of healthcare through the eye is gained by tracing its evolution from superstition towards the real. Yet, significant historical questions remain. Foundational theories of the external eye being representative of internal organs, including the Eye of Horus and the brain, the humoral color associations in Hippocratic texts and the somatic mappings of the eye in traditional Chinese medicine, remain insufficiently explored in terms of their empirical grounding and cross-cultural parallels. A provocative interpretive framework may be found in the writings of Moses Maimonides (Rambam), who suggested that certain ancient religious laws, such as the Levitical system of animal sacrifice, were not ends in themselves, but transitional pragmatic strategies designed to steer humanity away from destructive superstitious practices, such as ritual human sacrifice and cannibalism, toward ethical conduct and rational inquiry [(41), p. 526–31].

In this spirit, one might speculate that early figures such as the designers of the Eye of Horus and the authors of the *Hippocratic Corpus, Huang Di Nei Jing* and Ayurvedic texts similarly repurposed culturally powerful beliefs about the eye and internal health. Their aim may not have been to assert scientific accuracy, but rather to guide attention and thought away from victimhood and spiritual possessions toward agency and science, even while recognizing the limitations of their models. These foundational shifts helped pave the way for later scientific revolutions, ultimately enabling figures like al-Haytham, Galvani, and Harvey to develop empirically grounded models of physiology and perception. This hypothesis invites further interdisciplinary study. Investigating how early medical systems co-opted superstition as a conceptual bridge to rational thought may illuminate the historical progression from irrational belief systems towards evidence-based medicine.

There is a striking paradox in this analysis. While ancient thinkers may have used culturally prevalent believes about extramission, the external appearance of eyes, and internal health as vehicles to re-orient society towards rational thought and logic, the continued pursuit of these believes within the realm of science led to a convergence. The invention of the ophthalmoscope in 1851 confirmed that the eye does, in fact, reflect systemic health. Thus, an idea once rooted in superstition and later rational misconception evolved into a cornerstone of diagnostic medicine.

The historical evolution of healthcare through the eye highlights a recurring pattern that intuitive conclusions often precede accurate understandings. While modern imaging technologies have validated the eye as a diagnostic window, history cautions against assuming explanatory completeness. This perspective encourages contemporary researchers to pair high-dimensional ocular data with rigorous physiological validation, avoiding the pitfalls of earlier rational but incorrect models.

This narrative review has several limitations. First, it does not aim to provide exhaustive coverage of all global medical traditions, and many culturally rich systems are necessarily underrepresented. Second, historical interpretation relies on surviving texts and translations, which may reflect later editorial perspectives or incomplete records. Finally, the interpretive framework is intended to illuminate how different societies reasoned about the eye and internal health, rather than to assert direct influence between cultures. These limitations are inherent to historical synthesis and do not diminish the value of examining how enduring intuitions about the eye contributed to the evolution of modern ophthalmic diagnostics.

As ophthalmic imaging and analysis technologies continue to evolve, deeper insight is being gained into the promise of ocular biomarkers for predictive, preventive, and personalized medicine. With continued research and implementation, the eye may prove to be one of the most accessible and informative platforms for understanding and managing systemic disease. By bridging ancient perspectives with modern scientific inquiry, this work invites more historically informed and culturally responsive frameworks for delivering healthcare through the eye in today’s global context.

## Data Availability

The original contributions presented in the study are included in the article/supplementary material, further inquiries can be directed to the corresponding author/s.
